# Toll-Like Receptors in Esophageal Cancer

**DOI:** 10.3389/fimmu.2014.00200

**Published:** 2014-05-07

**Authors:** Joonas H. Kauppila, Katri S. Selander

**Affiliations:** ^1^Department of Pathology, University of Oulu, Oulu, Finland; ^2^Department of Surgery, University of Oulu, Oulu, Finland; ^3^Medical Research Center Oulu, Oulu, Finland; ^4^Oulu University Hospital, Oulu, Finland; ^5^Department of Hematology-Oncology, University of Alabama at Birmingham, Birmingham, AL, USA; ^6^Department of Pathology, Lapland Central Hospital, Rovaniemi, Finland

**Keywords:** Toll-like receptors, microbiome, esophageal cancer, esophageal adenocarcinoma, esophageal squamous cell carcinoma

## Abstract

Esophageal squamous cell carcinoma and esophageal adenocarcinoma are cancers of high mortality. EAC develops through Barrett’s esophagus (BE) and columnar dysplasia, preceded by gastro-esophageal reflux disease. The risk of esophageal squamous cell carcinoma is increased by smoking and alcohol consumption. New treatment options for esophageal cancer are desperately needed. Toll-like receptors (TLRs) play a central role in mammalian immunity and cancer. TLRs are activated by microbial components, such as lipopolysaccharide, flagellin, DNA, and RNA, as well as endogenous ligands, including heat-shock proteins and endogenous DNA. This review summarizes the studies on TLRs in esophageal squamous cell carcinoma and EAC. It has been shown that TLRs 1–10 are expressed in the normal esophagus. In esophageal squamous cell carcinoma, TLRs3, 4, 7, and 9 have been studied, showing associations to aggressive disease properties. In BE and EAC, only TLRs4, 5, and 9 have been studied. In the review, we discuss the implications of TLRs in esophageal cancer.

## Introduction

Toll-like receptors (TLRs) are evolutionarily conserved receptors of the innate immune system (1). The 13 TLRs that have been identified so far recognize their unique pathogen-associated molecular patterns (PAMPs), such as bacterial lipopolysaccharide (LPS) (TLR4), DNA (TLR9), or flagellin (TLR5) (1, 2). TLR stimulation induces down-stream activation of various signaling molecules and this ultimately results in the innate immune response, which also activates the adaptive immune system (1–3). The aim of this review is to explore the role and function of TLRs in esophageal adenocarcinoma (EAC) and in squamous cell carcinoma.

## Esophageal Cancer

Esophageal cancer is the eighth most common cancer in the world with estimated 482,000 new cases worldwide in 2008. The incidence of esophageal cancer was 70/100,000 in 2008 in the world. The majority of esophageal cancers are esophageal squamous cell carcinomas (ESCC), but the incidence of EAC is rising rapidly (4, 5).

As with oral squamous cell cancer, tobacco and alcohol, low socioeconomic status, poor oral health, and betel nuts, as well as the autoimmune polyendocrinopathy–candidiasis–ectodermal dystrophy (APECED)-syndrome have been listed as risk factors for ESCC (6–10). With regard to pathologic anatomy, esophagus could be considered as an extension of the oral cavity, as it is lined by squamous epithelium and it encounters swallowed oral bacteria before they enter the stomach.

For EAC, the most important risk factor is Barrett’s esophagus (BE), determined by columnar metaplastic cells, which replace the normal squamous epithelium after long-lasting gastro-esophageal reflux, or gastro-esophageal reflux disease (GERD). Patients with BE have a 30- to 125-fold risk for EAC compared to normal population (11, 12). A recent study, however, concluded that only 0.12% of patients with BE develop EAC (13). Other minor risk factors include obesity, smoking, hiatal hernia, and low socioeconomic status (10, 14–18). Furthermore, both types of esophageal cancers develop through dysplasia to cancer via genetic alterations (19, 20).

The 5-year survival rate for esophageal cancer varies between 10 and 16% (4). After esophageal resection, the 5-year survival rate was 20.6% in a meta-analysis of Western population (21). Furthermore, these cancers are often diagnosed late because at the time of the diagnosis, more than half of the patients have an inoperable disease (22).

The most important prognostic determinant for both esophageal cancers is the WHO TNM-classification (23). The histologically defined grade of differentiation is also a predictor of prognosis (24).

## Toll-Like Receptors in Normal Esophagus

Esophageal epithelial cells have been shown to express TLRs. The human esophageal epithelial cell-line TE-1 was shown to express TLRs2, 3, 4, and 7, with up-regulation of beta-defensin 2 as a response to stimulation with their cognate, synthetic ligands (25). In 2009, Lim and colleagues demonstrated the expression of TLRs 1–10, but not TLR4 at the mRNA level in the normal human esophageal epithelial cell-line EPC-2. Furthermore, they demonstrated TLR1, 2, 3, and 5 mRNA expression in biopsies taken from esophageal mucosa. IL-8 was up-regulated in the EPC-2 cells by stimulation of the respective TLR-ligands. TLR3 stimulation was the most effective in inducing IL-8 expression synergistically with TLR2 and this effect was dependent on NF-kB activation (26).

TLR3 was later demonstrated also to mediate the induction of IL-8 mRNA via NF-kB by necrotic cell supernatants in the EPC-2 cells (27). TLR2 and TLR3 protein expression was demonstrated in esophageal epithelial cells, but not in cultured primary esophageal epithelial cells (28). The expression of TLR3, 4, 5, 7, and 9 proteins in normal esophagus has been characterized using immunohistochemistry in clinical samples (29–31). These studies have demonstrated that TLRs 1–10 are expressed in normal esophagus.

## Toll-Like Receptors and Esophageal Squamous Cell Carcinoma

Esophageal squamous cell carcinoma develops to squamous epithelium via dysplasia. A variety of TLRs, including TLR3, 4, 7, and 9, have been shown to be overexpressed in esophageal squamous cell carcinoma, when compared to normal esophagus (30, 31). We demonstrated an increased TLR9 expression in esophageal squamous dysplasia and in squamous cell carcinoma, suggesting a possible role for TLR9 in esophageal carcinogenesis (31).

High TLR3, 4, and 9 expression in esophageal squamous cell carcinoma cells have been associated with lymph node metastasis and TLR7 and 9 expression to worse histological grade (30, 31). TLR9 expression in the fibroblastoid cells of the tumor was, however, associated with decreased invasion depth and a smaller prevalence of lymph node metastasis at the time of diagnosis (30). TLR4 stimulation by LPS has been shown to increase migration and adhesive properties of esophageal squamous cell carcinoma cells via p38 and selectin (32). No studies thus far have evaluated the anti-cancer efficacy of TLR-agonists or inhibitors in the treatment of ESCC.

## Toll-Like Receptors, Barrett’s Esophagus, and Esophageal Adenocarcinoma

Esophageal adenocarcinoma is developed through the metaplasia–dysplasia–carcinoma sequence. Normal or inflamed esophageal epithelium is believed to transform to BE or columnar metaplasia through continuous exposure to acidic gastric contents, but also transformation of esophageal microbiome occurs during these changes (33, 34).

It was shown in BE and in normal human esophageal cell lines, that stimulation of TLR4 with LPS resulted in NF-κB activation and an increase of IL-8 secretion, this response was more significant in BE. *Ex vivo* culture demonstrated increased cyclo-oxygenase-2 (COX-2) activation by LPS stimulation of TLR4 in BE (35). TLR5 was recently analyzed in the metaplasia–dysplasia–adenocarcinoma sequence, with high expression potentially differentiating between BE and columnar dysplasia (29).

The increased expression of TLR5 and 9 has been shown in EAC. TLR5 expression had no associations to clinico-pathological variables or prognosis, but TLR9 expression was associated with metastasis, poor grade of differentiation and poor prognosis in EAC (29, 36). Stimulation of EAC cells with CpG-oligonucleotides that either have the physiological phosphodiester DNA-backbone or the nuclease-resistant phosphothioate backbone, induced cellular invasion and matrix metalloproteinase-9 and -13 mRNA expression (37).

At the current moment, there are no published clinical studies on TLRs in EAC.

## Toll-Like Receptor Genetics and Esophageal Cancer

Genetic studies have been performed on Toll-like receptor polymorphisms in esophageal cancer. Unlike in gastric cancer, polymorphisms in *TLR4* + *896A* > *G* and *TLR9-1237T/C* genes were not associated to esophageal cancer risk (38, 39). However, genetic up-regulation of CD14, a co-receptor of TLR4, was observed in families with history of esophageal cancer (40).

## Discussion

The treatment of esophageal cancer is overshadowed by its poor prognosis. New options for early diagnosis and treatment are desperately needed. The esophageal epithelium encounters bacteria from oral cavity and in the case of reflux disease, also from the stomach and possibly also from the duodenum. TLRs act by recognizing bacteria-derived molecular patterns which results in a pro-inflammatory reaction in the epithelium.

The role of TLRs in esophageal cancer has been studied sparsely. However, there is evidence that the function of TLRs is pro-carcinogenic and pro-inflammatory as the overexpression of many of the TLRs have been linked with esophageal cancer and with poor prognosis. Inflammation is a known important factor in the pathogenesis of various cancers. It was demonstrated by Yang et al. that the microbiome of distal esophagus frequently undergoes changes during esophagitis and BE. During these processes, the microbiome is switched from aerobic to gram-negative anaerobic bacteria (33, 34). This finding together with abnormal TLR expression, particularly those of TLRs4, 5, and 9, in esophageal cancer supports the hypothesis of bacteria contributing to the carcinogenesis of esophageal cancer. These findings further suggest that TLRs may be important mediators for bacteria in oncogenesis (37, 40, 41).

**Figure 1:**
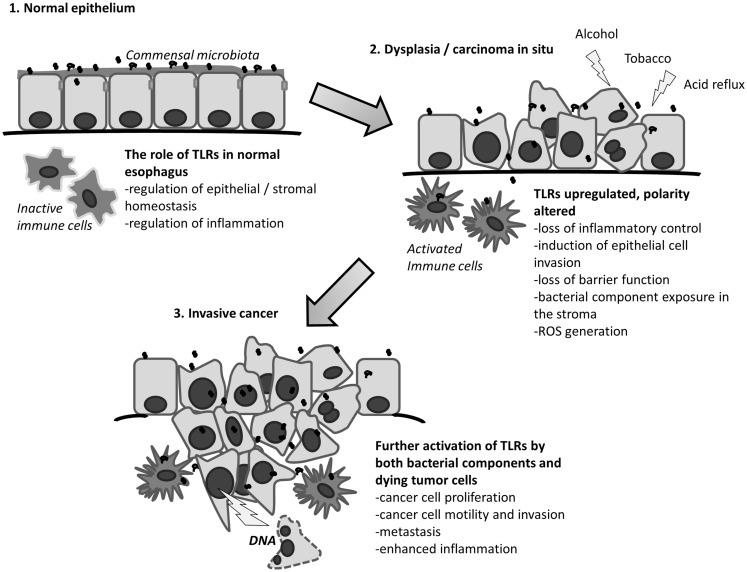
**The proposed role of Toll-like receptors in esophageal cancer**. Modified from Kauppila et al. (37).

In addition to microbes, TLRs can also detect molecular patterns that are derived from the host itself. TLRs3, 4, and 9 are known to be activated by endogenous ligands from dead or damaged host cells (42, 43). The combination of cellular damage by alcohol, tobacco, and acidic contents of the stomach results in the loss of epithelial wall integrity, through epithelial cell death and by disruption of the cell-to-cell contacts. Especially TLR3 and TLR9 (but also other TLRs) can recognize particles from dead cells (43). This can result in an inflammatory wound reaction through the activation of interleukins, NF-kB, and matrix metalloproteinases. This wound reaction could facilitate the passage of bacteria through epithelium and result in the loss of host-microbiome homeostasis, further leading to abnormal activation of for example TLR2, 4, 5, and 9 by bacterial components. Inflammation and wound reaction then could produce a vicious cycle of cellular damage, which might be a major player in esophageal metaplasia and carcinogenesis. This role of bacteria and TLR4 in genesis of BE has been discussed earlier by Yang et al. (33). Cell-to-cell junctions become dysfunctional in exogenous damage to the epithelium as discussed earlier. Thus, a similar effect can also be observed in dysplasia and cancer (44). This dysfunction may lead to Toll-like receptor activation in cancer by exogenous and endogenous ligands. The hypothesis is summarized in Figure 1.

Finally, Toll-like receptor expression is up-regulated in both squamous cell carcinoma and adenocarcinoma of the esophagus. This may indicate that cancer cells are sensitized to bacteria- and host-derived ligands. Poor prognosis in strongly TLR-expressing tumors could then be an indicator of increased level of tumor–stroma interaction.

## Conclusion

There seems to be a connection between TLRs and esophageal cancer development. The fact that bacterial flora changes during esophageal metaplasia and inflammation, as well as observed up-regulation of TLRs in esophageal cancers support the hypothesis that bacteria as well as TLRs have a role in esophageal cancer.

## Conflict of Interest Statement

The authors declare that the research was conducted in the absence of any commercial or financial relationships that could be construed as a potential conflict of interest.
